# Apolipoprotein M and sphingosine-1-phosphate complex alleviates TNF-α-induced endothelial cell injury and inflammation through PI3K/AKT signaling pathway

**DOI:** 10.1186/s12872-019-1263-4

**Published:** 2019-12-02

**Authors:** Yang Liu, Li Tie

**Affiliations:** 1 International Medical Center, Geriatric Department, National Clinical Research Center for Geriatric Disorders, Xiangya Hospital, Central South University, No 87 of Xiangya Road, Kaifu District, Changsha, Hunan 410008 People’s Republic of China; 2 Department of Cardiology, Changsha Central Hospital, Changsha, Hunan 410008 People’s Republic of China

**Keywords:** Pyroptosis, TNF-α, ApoM, S1P, PI3K/AKT, Inflammation, Human umbilical vein endothelial cells

## Abstract

**Background:**

In spite of the important role of Apolipoprotein-M (ApoM) and Sphingosine-1-Phosphate (S1P) played in atherosclerosis (AS), there was few related research reporting ApoM and S1P complex (ApoM-S1P) on biological activities of human umbilical vein endothelial cells (HUVECs). In this study, we explored the effect and mechanism of ApoM-S1P on TNF-α-induced inflammation in HUVECs.

**Methods:**

TNF-α was utilized to induce HUVEC injury and inflammation. After HUVECs were treated with antagonists of ApoM, S1P, ApoM + S1P, and ApoM + S1P + S1PR, calcein-acetoxymethyl ester was employed for the assessment of the adhesion of HUVECs to THP-1, immunofluorescence for the observation of caspase-1expression in HUVECs, reactive oxygen species (ROS) kit for the detection of ROS level in HUVECs. The impact of TNF-α, ApoM, S1P and S1PR antagonists on inflammatory response, pyroptosis and adhesion of THP-1 monocytes to HUVECs were determined by detecting expressions of pyroptosis related proteins (IL-1β, IL-18, ASC, NLRP3 and caspase-1), inflammatory cytokines (IL-6 and IL-10), adhesion molecules (E-selectin, ICAM-1, and VCAM-1) and p-PI3K/p-AKT by qRT-PCR and Western blot, as well as by ELISA.

**Results:**

TNF-α could increase adhesion of THP-1 monocytes to HUVECs and induce inflammatory response and pyroptosis in HUVECs, indicated by up-regulated expressions of E-selectin, ICAM-1, VCAM-1, IL-1β, IL-18, caspase-1, ASC, NLRP3, and IL-6, and down-regulated expression of IL-10. Co-treatment of ApoM-S1P on TNF-α treated HUVECs could protect HUVECs from injury and inflammation, evidenced by the attenuation of expressions of pyroptosis related proteins, inflammatory cytokines, and adhesion molecules, as well as the augment of PI3K and AKT phosphorylation. JTE-013, an antagonist of S1PR2, could reverse the amelioration of ApoM-S1P on pyroptosis and inflammation of HUVECs, indicating that ApoM-S1P could bind to S1PR2 to protect HUVECs from injury and inflammation through activating PI3K/AKT pathway.

**Conclusion:**

ApoM-S1P could attenuate TNF-α induced injury and inflammatory response in HUVECs by binding to S1PR2 to activate PI3K/AKT pathway.

## Background

Atherosclerosis (AS) is a widespread disease that causes morbidity and mortality, which could deteriorate into circulatory problems including cerebrovascular disease and coronary disease [1, 2]. Despite the great advancement on both basic and clinical researches, the incidence of AS has been increased dramatically in recent decades and has resulted in millions of deaths around the world each year [3, 4]. AS is characterized by the sedimentation of fibrous tissues and lipids in the intima of elastic arteries, with consequent formation of thrombus and structural damage caused by thickening and hardening of the vessel walls [5]. Previous study has unraveled that cell death and inflammation play critical roles in various stages of AS [6]. Typically, cell death is especially attributed to apoptosis and necrosis, nevertheless, other forms of cell death including pyroptosis has also been discovered [7]. Pyroptosis is a new type of pro-inflammatory programmed cell death different from apoptosis and necrosis, which can lead to cytokines release and activate pro-inflammatory immune cell mediators [8, 9]. However, the roles of pyroptosis and its potential relationship with AS as well as the underlying mechanisms remained inadequately investigated.

Sphingosine-1-phosphate (S1P) is a membrane-derived sphingolipid that regulates a variety of cellular processes including migration, growth, inflammation, survival, and angiogenesis, and is considered as a key regulator of AS physiology and pathophysiology [10, 11]. Up-regulated expression of pro-inflammatory cytokine IL-6 is proved to increase S1P at cellular level, which consequently results in elevated expression of S1P receptor 1 (S1PR1) in sickle cell disease mice models [12]. S1P signaling exerts a critical impact on lymphocyte transportation, blood vessel development, and neurogenesis and S1P receptor antagonist has been approved for the control of autoimmune neuroinflammation in patients with multiple sclerosis [13]. Inhibition of endothelial S1PR2 signaling attenuates endothelial senescence associated with functional impairment and endothelial activation induced by atherogenic stimulation [14]. As a carrier for S1P, apolipoprotein M (ApoM) is one of the apolipoprotein components of high-density lipoprotein (HDL), processing atheroprotective function by regulating cholesterol efflux [15, 16]. The implication of ApoM and S1P in AS has been evidenced in rodent models [17]. Nevertheless, the impact of ApoM-S1P complex (ApoM-S1P) in AS is presently controversial [18]. Data in recent study demonstrated that pyroptosis may be associated with AS by affecting the stability of atherosclerotic lesion [5]. Considering the important role of ApoM-S1P in AS, it is possible that ApoM-S1P may regulate pyroptosis through certain pathway in AS, while no such studies verified our hypothesis. Here, we assessed the impact of ApoM-S1P on injury and inflammation of HUVECs and its possible mechanism. In this study, we discovered that ApoM-S1P could inhibit TNF-α-induced inflammation and pyroptosis of HUVECs via binding S1PR2 to activate the PI3K/AKT pathway.

## Methods

### Cell culture

Human umbilical vein endothelial cells (HUVECs) and THP-1 monocytes were acquired from Type Culture Collection of the Chinese Academy of Sciences (Shanghai, China). HUVECs were nurtured in DMEM medium comprising 1% penicillin/streptomycin and 10% fetal bovine serum (FBS). When HUVECs were fused into monolayer cells, they were passaged with 0.125% pancreatin-0.02% EDTA digestion. The 3-5th generations of HUVECs were used for experiments. HUVECs were starved for 10 h in DMEM medium containing 1% FBS before being pre-treated with tumor necrosis factor-α (TNF-α, 50 ng/mL, Peprotech, Rocky Hill, NJ, USA) for 6 h [19, 20]. THP-1 monocytes were incubated in RPMI-1640 medium, supplemented with 1% penicillin and streptomycin, 10% FBS, 1% L-glutamine, 1% HEPES buffer and 0.05 mM β-mercaptoethanol. Both HUVECs and THP-1 monocytes were nurtured at 37 °C in a 5% CO_2_ incubator.

### Load ApoM with S1P

An eight-folds of S1P (Cayman, Ann Arbor, MI, USA) was added to ApoM (Abnova, Taiwan, China) and incubated for 30 min at room temperature to load ApoM with S1P. Unbound S1P was removed using a desalting PD10 column (GE Healthcare, Beijing, China) after the mixture was incubated with serum-free M200 medium. Then ApoM-S1P was diluted to 0.5 μM and stored at  -20 °C.

### THP-1-HUVECs adhesion assays

Before adhesion assays, HUVECs were pretreated with 50 ng/mL of TNF-α for 6 h. Then HUVECs were treated with ApoM (1.0 μM), S1P (1.0 μM), or ApoM-S1P for 24 h [21] and grouped into TNF-α group, Control group, TNF-α + ApoM group, TNF-α + S1P group, and TNF-α + ApoM + S1P group. Antagonists for S1P receptors (S1PR1, S1PR2 and S1PR3) were added in TNF-α stimulated HUVECs for 30 min before cells were subjected to ApoM-S1P treatment. According to antagonist treatment, cells were grouped into TNF-α + ApoM + S1P + W146 group, TNF-α + ApoM + S1P + JTE-013 group, and TNF-α + ApoM+S1P + CAY10444 group, respectively.

THP-1 monocytes were labeled with 5 μmol/L calcein-acetoxymethyl ester (Thermo Fisher Scientific, Waltham, MA, USA) and cultured in the incubator for 30 min. The labeled cells were washed twice with PBS and re-suspended in serum-free medium. Then culture medium of HUVECs was removed. The resuspensions of labeled THP-1 monocytes were added onto monolayers of HUVECs. After 1 h incubation, the plates were rinsed twice with medium without serum. The adherent THP-1 monocytes on HUVECs were counted with a fluorescence microscope (Olympus IX71, Tokyo, Japan). The culture dish was divided into four quadrants according to its diameter. For each quadrant, one 200 × visual field in the middle of the quadrant was chosen for analysis. The average number of the four quadrants was identified as the number of adherent THP-1 monocytes.

### Quantitative real-time polymerase chain reaction (qRT-PCR)

Total RNA of HUVECs was extracted by RNeasyPlus Micro (Qiagen, dusseldorf, Germany). The ratio of OD_260 nm_ to OD_280 nm_ was measured to quantify and test the purity of RNA. Reverse Transcription of RNA into cDNA was conducted by Reverse Transcription System (Promega, Madison, WI, USA). The mRNA expression levels of E-selectin, ICAM-1, VCAM-1, IL-1β, IL-18, caspase-1, ASC, NLRP3, IL-6, and IL-10 were detected by qRT-PCR using SYBR Premix ExTaq (Takara Bio, Inc., Otsu, Japan). GAPDH was regarded as internal reference. All tests were conducted in triplicates. All data and figures were depicted as the mean ± standard deviation (SD), and data analysis was employed the 2^-ΔΔCt^ method [22]. The amplified primer sequences of each gene and its primers are shown in Table 1.

**Table 1:** Primer sequences information

Name	Primer
E-selectin	F: 5′-ATGTGAAGCTGTGAGATGCG-3’
	R: 5′-CCACTGCAGCTCATGTTGAT-3’
ICAM-1	F: 5′-AGACATAGCCCCACCATGAG-3’
	R: 5′-CAAGGGTTGGGGTCAGTAGA-3’
VCAM-1	F: 5′-TCGCTCAAATCGGTGACTCC-3’
	R: 5′-ACTTCGTTCCAGCTTCCCAG-3’
IL-1β	F: 5′-GCAGGCAGTATCACTCATTGTGG-3’
	R: 5′-GAGTCACAGAGGATGGGCTCTTC-3’
IL-18	F: 5′-ACCCCAGAAGAGAGGGAGTC-3’
	R: 5′-GTAGATGGTGGAATCGGCGT-3’
caspase-1	F: 5′-CACGAGACCTGTGCGATCAT-3’
	R: 5′-CTTGAGGGAACCACTCGGTC-3’
ASC	F: 5′-GTCCAGGTTCCGCCCC-3’
	R: 5′-AACTTCTTGAGCTCCTCGGC-3’
NLRP3	F: 5′-AAACGACCTTCATCCCCACC-3’
	R: 5′-CAGGACTGCCCTCCTCTAGT-3’
IL-6	F: 5′-GAGGAGACTTCACAGAGGATACCAC-3’
	R: 5′-TTGCCATTGCACAACTCTTTTC-3’
IL-10	F: 5′-CCAACGAACCGTCCCTGTTA-3’
	R: 5′-TCGGAGTTGTGAGCTTCCAC-3’
GAPDH	F: 5′-TCTTGTGCAGTGCCAGCCT-3’
	R: 5′-TGAGGTCAATGAAGGGGTCG-3’

Notes: *F* Forward primer, *R* Reversed primer

### Western blotting

Cell lysates were made from HUVECs using RIPA buffer comprising phosphatase inhibitors (Cayman Chemical, Ann Arbor, MI, USA) and Halt protease inhibitors (Pierce, Rockford, IL, USA). Bradford assay was adopted to test the concentration of proteins. The cell lysates were mixed with loading buffer and separated on a 10% SDS polyacrylamide gels. The proteins on gels were then transferred onto PVDF membrane. The membranes were blocked in TBST containing 0.05 g/mL BSA for 1 h. After blocking, the membranes were incubated overnight at 4 °C with primary antibodies (abcam, san francsico, USA), rabbit-derived GAPDH (1:10000, ab181602), E-selectin (1:200, ab18981), VCAM-1 (1:2000, ab134047), ICAM-1 (1:2000, ab53013), caspase-1(1:500, ab138483), NLRP3 (1:500, ab214185), ASC (1:1000, ab155970), PI3K (1:1000, ab191606), p-PI3K (1:1000, ab182651), AKT (1:10000, ab179463), p-AKT (T308, 1:1000, ab38449) and p-AKT (Ser473,1:2000, 4060 T, Cell Signaling Technology, Boston, USA). The membranes were washed in TBST and then incubated at room temperature for 2 h with goat anti-rabbit IgG (1:5000, Beijing ComWin Biotech Co., Ltd., Beijing, China). The membranes were then rinsed three times with TBST. ECL luminescent solution was used before a chemiluminecence imaging analysis system (GE Healthcare, USA) was applied for observation.

### Enzyme-linked Immunosorbent assay (ELISA)

The supernatants from untreated and treated HUVECs were centrifuged (300 g, 5 min) and cell-free supernatants were frozen at  -80 °C until usage. The assays were conducted utilizing commercial ELISA kits (abcam, san francsico, USA) in duplicates, according to the manufacturers’ instructions. The microplate absorbance reader (Tecan, Groening, Austria) was employed to measure the absorbance at 450 nm. Standard curves were applied to calculate the concentrations of analytes.

### Immunofluorescence

HUVECs were seeded in 24-well plates at the density of 5 × 10^3^ cells/well, followed by fixation in 4% paraformaldehyde for 15 min, permeation with 0.5% triton-X-100 for 20 min, and incubation with appropriate concentration of primary antibody of caspase-1 (1:500, ab1872, abcam, san francsico, USA) overnight at 4 °C. After that, sections were incubated with secondary antibody of Alexa Fluor 488-labeled goat anti-rabbit IgG (ab150077, 1:2000, abcam, MA, USA) for 45 min avoiding light, prior to PBS rinsing. Then cells were stained with 0.5 μg/mL of DAPI for 15 min avoiding light, and washed with PBS. Subsequently, sections were sealed and observed under a fluorescence microscope (Olympus IX71, Tokyo, Japan).

### Detection of reactive oxygen species (ROS)

The ROS was measured using DCF-DA kit (BestBio, Shanghai, China) according to the manufacturer’s instructions. Cells (1 × 10^7^/mL) were labeled with 10 μM of fluorescent probe DCFH-DA, followed by incubation in an incubator for 20 min at 37 °C, and serum-free cell culture solution washing for three times. The fluorescence activity was analyzed by fluorescence microscopy with an excitation wavelength of 488 nm and an emission wavelength of 520 nm. The amount of intracellular ROS was proportional to the fluorescence intensity of DCF, and the fluorescence intensity indicated the ROS level.

### Statistical analysis

All data were analyzed using SPSS 17.0 software (SPSS, Chicago, IL, USA) and GraphPad Prism 6.0. *T* test was used for comparison between two groups, and differences among groups were analyzed using One-way analysis of variance. *P* < 0.05 was regarded as a statistically significant difference.

## Results

### TNF-α enhances the adhesion of HUVECs and induces pyroptosis

**Fig. 1:**
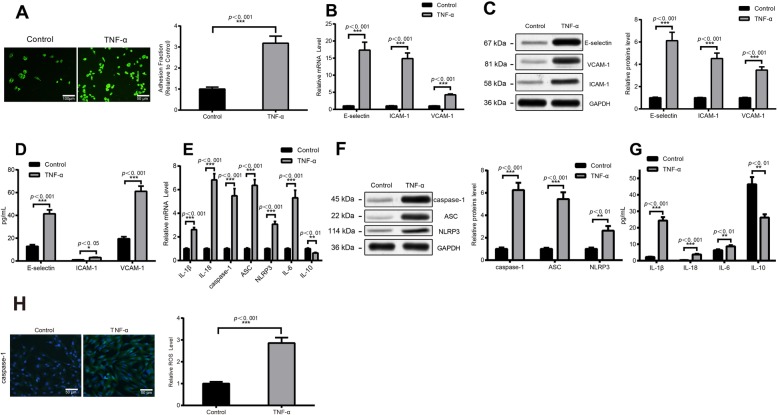
TNF-α enhances the adhesion function and induce pyroptosis of HUVECs Notes: After stimulating HUVECs with TNF-α treatment, fluorescence microscope was applied to observe the number of adhesion of THP-1 to HUVECs (200 ×) (**a**); The effect of TNF-α on mRNA and protein levels of ICAM-1, E-selectin, and VCAM-1 were inspected by qRT-PCR (**b**), Western blot (**c**) and ELISA (**d**); The effect of TNF-α on pyroptosis was determined by qRT-PCR (**e**), western blot (**f**) and ELISA (**g**) to detect the expression levels of IL-1β, IL-18, caspase-1, ASC, NLRP3, IL-6, and IL-10; The expression of caspase-1 was performed by immunofluorescence (**h**), and ROS level was measured by DCF-DA kit (I); Data were presented as mean ± SD, ^**^P < 0.01 ^***^P < 0.001 compared to the Control group; HUVECs, human umbilical vein endothelial cells; SD, standard deviation

In the early stages of AS, endothelial cell function is impaired, and the expression of adhesion molecules is significantly increased, thereby recruiting pro-inflammatory immune cells to adhere to the vascular wall [23]. In this study, we discovered that the number of THP-1 monocytes adhering to the surface of HUVECs in the TNF-α group was significantly increased compared with the Control group (Fig. 1a), and up-regulate the mRNA and protein levels of adhesion molecules E-selectin, ICAM-1, VCAM-1 (*P* < 0.001) (Fig. 1b-c). ELISA illustrated that the contents of three adhesion molecules in TNF-α group were higher than those in the Control group (*P* < 0.05) (Fig. 1d). All these stated that TNF-α could enhance the adhesion function of HUVECs and induce the generation of adhesion molecules.

Pyroptosis is a more recently identified pathway of programmed cell death and accompanied with the severe inflammation response [24]. The detection on effect of TNF-α treatment on pyroptosis of HUVECs displayed that the mRNA levels of caspase-1, ASC, NLRP3, IL-1β and IL-18 in TNF-α group were higher than those in Control group, while anti-inflammatory cytokine were lower than Control group (*P* < 0.01) (Fig. 1e). Consistent expression pattern was found on protein expressions of pyroptosis and inflammation related factors (*P* < 0.05) (Fig. 1f). ELISA results manifested that compared with Control group, the contents of IL-1β, IL-18, and pro-inflammatory cytokine IL-6 in TNF-α group were increased, while the content of anti-inflammatory cytokine IL-10 was decreased (*P* < 0.01) (Fig. 1g). Subsequently, immunofluorescence was utilized to inspect the expression of caspase-1 in HUVECs. The results manifested that TNF-α group had higher caspase-1 expression than the Control group (Fig. 1h). Meanwhile, DCF-DA kit detection results described that there was elevated ROS level in TNF-α group in comparison to the Control group (*P* < 0.001) (Fig. 1i). These indicated that TNF-α could enhance inflammation and promote pyroptosis of HUVECs.

### ApoM-S1P reduces cell adhesion and pyroptosis induced by TNF-α

**Fig. 2:**
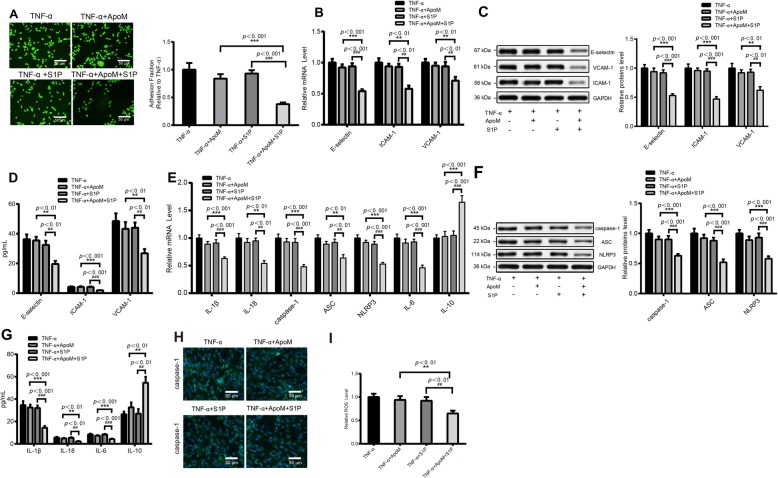
ApoM-S1P dramatically reduces TNF-α induced cell adhesion and pyroptosis Notes: After TNF-α stimulated HUVECs were treated with ApoM-S1P, the adhesion of THP-1 monocytes to HUVECs were counted under a fluorescence microscopy (200 ×) (**a**); The impact of ApoM-S1P on expressions of adhesion molecules E-selectin, ICAM-1, VCAM-1 were determined by qRT-PCR (**b**), Western blot (**c**) and ELISA (**d**); the expressions of pyroptosis and inflammatory related cytokines IL-1β, IL-18, caspase-1, ASC, NLRP3, IL-6, and IL-10 were verified after cells were subjected to ApoM-S1P treatment by qRT-PCR (**e**), Western blot (**f**) and ELISA (**g**); Immunofluorescence was used to assay caspase-1 expression (**h**), and ROS level evaluated by DCF-DA kit (**i**); Data were presented as mean ± SD, ^**^P < 0.01 ^***^P < 0.001 compared to the TNF-α + ApoM group; ^##^*P* < 0.01 ^###^*P* < 0.001 compared to TNF-α + S1P group; HUVECs, human umbilical vein endothelial cells

ApoM, a specific S1P chaperone, is a novel apolipoprotein associated with anti-inflammatory functions [25]. Study has illustrated the effect of ApoM-S1P in mediating anti-inflammatory effects on endothelial cells [23]. Hence, we investigated whether ApoM-S1P treatment could affect TNF-α-induced adhesion and pyroptosis of HUVECs. Fluorescence microscope observed that compared with the TNF-α + ApoM group or TNF-α + S1P group, TNF-α + ApoM + S1P group had suppressed adhesion quantity of THP-1 monocytes (*P* < 0.001) (Fig. 2a). In addition, ApoM-S1P treatment could substantially decrease the protein expression levels of VCAM-1, E-selectin, and ICAM-1 (*P* < 0.01). While there were no significant changes of adhesion quantity of THP-1 monocytes in both TNF-α + ApoM group and TNF-α + S1P group in comparison to that in TNF-α group (*P* > 0.05) (Fig. 2b-d). In addition, qRT-PCR results explained that TNF-α + ApoM + S1P group had a suppressed the mRNA levels of IL-1β, IL-18, caspase-1, ASC, NLRP3 and IL-6, and an elevated mRNA level of IL-10 than TNF-α + ApoM group or TNF-α + S1P group (*P* < 0.01) (Fig. 2e). The results obtained by Western blot and ELISA supported the protective effect of ApoM + S1P on pyroptosis and inflammation of HUVECs (*P* < 0.01) (Fig. 2f-g). In addition, compared with TNF-α group, no significant difference was revealed in TNF-α + ApoM group and TNF-α + S1P group (*P* > 0.05) (Fig. 2e-g). Then immunofluorescence was employed to assay caspase-1 expression in HUVECs. The results presented that TNF-α + ApoM+S1P group had heightened caspase-1 expression compared with the TNF-α + ApoM group or TNF-α + S1P group (Fig. 2h), whereas caspase-1 expression showed no obvious difference in cells treated with ApoM or S1P alone when compared with cells in TNF-α group (TNF-α + ApoM group or TNF-α + S1P group vs TNF-α group; *P* > 0.05) (Fig. 2h). Meanwhile, DCF-DA kit detection results displayed that TNF-α + ApoM+S1P group had elevated ROS level in comparison to TNF-α + ApoM group or TNF-α + S1P group (*P* < 0.001) (Fig. 2i), and there was no significant difference of ROS level in either TNF-α + ApoM group, or TNF-α + S1P group when compared with TNF-α group (*P* > 0.05). These results indicated that ApoM-S1P can observably reduce TNF-α-induced adhesion and cell pyroptosis.

### ApoM-S1P inhibits TNF-α-induced adhesion and pyroptosis via S1PR2

**Fig. 3:**
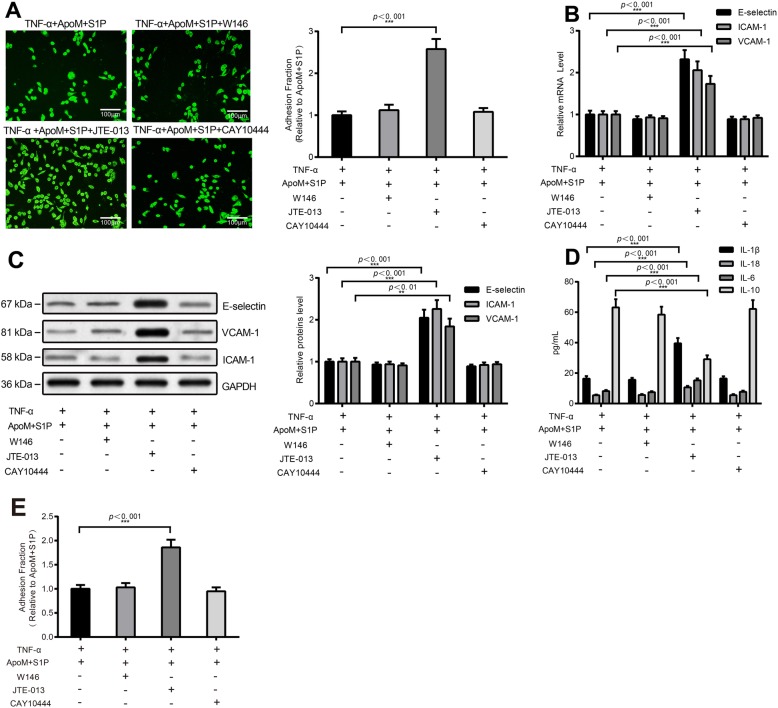
ApoM-S1P inhibits TNF-α induced adhesion and pyroptosis through binding to S1PR2. Notes: To explore the downstream mechanism of ApoM-S1P on attenuation of pyroptosis and inflammatory response of HUVECs, S1PR2 antagonist was added in TNF-α stimulated HUVECs before cells were subjected to ApoM-S1P treatment. Fluorescence microscope was used to count the number of adhesion of THP-1 monocytes (200 ×) (**a**); The mRNA and protein levels of E-selectin, ICAM-1, and VCAM-1in HUVECs were detected by qRT-PCR (**b**) and Western blot (**c**); ELISA was utilized to test the contents of IL-1β, IL-18, IL-6, and IL-10 (**d**); DCF-DA kit for inspection of ROS level (**e**); Data are displayed as mean ± SD, ^**^P < 0.01 ^***^P < 0.001 compared to the TNF-α + ApoM + S1P + JTE-013 group; HUVECs, human umbilical vein endothelial cells; SD, standard deviation

To further investigate the mechanism of ApoM+S1P inhibition on inflammatory factors and adhesion molecules, TNF-α-induced HUVECs were pretreated with specific S1PR1 antagonist W146, S1PR2 antagonist JTE-013, or S1PR3 antagonist CAY10444 for 30 min before ApoM-S1P treatment. Compared with TNF-α + ApoM + S1P group, TNF-α + ApoM + S1P + JTE-013 group had an elevated adhesion quantity of THP-1 monocytes, while TNF-α + ApoM + S1P + W146 group and TNF-α + ApoM + S1P + CAY10444 group showed no difference with TNF-α + ApoM+S1P group (Fig. 3a). Detection on adhesion molecules demonstrated that E-selectin, ICAM-1 and VCAM-1 were increased after JTE-013 treatment in comparison to TNF-α + ApoM + S1P group (Fig. 3b-c). Results also demonstrated that JTE-013 could reverse the protective impact of ApoM + S1P on pyroptosis of HUVECs, evidenced by increased IL-1β, IL-18 and IL-6, and decreased expression of IL-10 (*P* < 0.001) (Fig. 3d). No substantial difference was uncovered for TNF-α + ApoM + S1P + W146 group and TNF-α + ApoM + S1P + CAY10444 group when compared with TNF-α + ApoM + S1P group. Subsequently, DCF-DA kit detection results manifested that JTE-013 group had elevated ROS level in comparison to TNF-α + ApoM + S1P group (*P* < 0.001) (Fig. 3e), and there was no markedly difference of ROS level between W146 group, CAY10444 group, and TNF-α + ApoM + S1P group (*P* > 0.05). These results indicate that ApoM-S1P inhibits TNF-α-induced adhesion and cell pyroptosis by binding to S1PR2.

### ApoM-S1P plays a protective role through PI3K/AKT signaling pathway

**Fig. 4:**
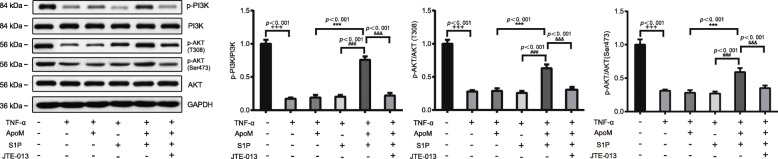
ApoM-S1P activates PI3K/AKT signaling pathway to attenuate pyroptosis and inflammatory response of HUVECs. Note: The detection of phosphorylation level of PI3K and AKT by Western blot. Data were shown as mean ± SD, ^**^P < 0.001 compared to the Control group, ^***^P < 0.001 compared to the TNF-α + ApoM group, ^***^P < 0.001 compared to TNF-α + ApoM+S1P group; HUVECs, human umbilical vein endothelial cells; SD, standard deviation

Previous evidence has demonstrated that PI3K/AKT is a downstream signaling pathway of S1P [26]. Therefore, we hypothesized that ApoM+S1P may exert its protective role in AS though PI3K/AKT signaling pathway. In this regards, we detected the effect of ApoM, S1P or/and SIPR2 antagonist JTE-013 on phosphorylation level of PI3K and AKT with the application of Western blot. The analysis revealed that TNF-α group had a lower phosphorylation level of PI3K and AKT than Control group. TNF-α + ApoM + S1P could partially restore the phosphorylation level of PI3K and AKT when compared with TNF-α group, while SIPR2 antagonist JTE-013 could inhibit the phosphorylation level of PI3K and AKT in comparison to ApoM-S1P treatment alone (*P* < 0.001). The phosphorylation level of PI3K and AKT in TNF-α + ApoM + S1P group was substantially higher than those in TNF-α + ApoM group and TNF-α + S1P group (*P* < 0.001) (Fig. 4). These indicated that ApoM-S1P could reverse the inhibitory effect of TNF-α on PI3K/AKT signaling pathway via mediating SIPR2.

## Discussion

TNF-α is a critical inflammatory factor that has been displayed to cause the interaction between vascular endothelial cells and invading monocytes [20]. TNF-α also plays a crucial role in atherosclerotic inflammatory cascade through up-regulation expression of adhesion molecule, activation of endothelial cell, and remodeling of vascular [27]. In the current study, HUVECs was firstly stimulated by TNF-α and then subjected to ApoM, SIP, or ApoM-S1P treatment to observe the changes on expression levels of cell adhesion, inflammation, and pyroptosis related factors. Our study displayed that ApoM-S1P could markedly down-regulate the expression of the pro-inflammatory cytokine, adhesion molecules, and pyroptosis related proteins by binding to S1PR2 so as to active the PI3K/AKT signaling pathway, thereby inhibiting the inflammatory response and pyroptosis of TNF-α-induced HUVECs.

ApoM-S1P has recently been proved to possess unique anti-inflammatory effects [18]. In this study, we discovered that ApoM-S1P exerts a crucial prestige on inflammatory response and pyroptosis of TNF-α induced HUVECs. Previous results support the implication of ApoM-S1P in various diseases. For example, ApoM-S1P has a major influence on diet-induced obesity and postprandial triglyceride metabolism [28]. Furthermore, ApoM-S1P is a pivotal element of anti-apoptotic activity of HDLs, which can promote the optimization of endothelial function [29]. Another research illustrated that ApoM-S1P is a therapeutic target for endothelial barrier dysfunction [30]. In addition, AS is reported to be associated with inflammatory processes in the endothelial cells of the vessel wall [31]. Consistent with results from previous study, we found ApoM-S1P could attenuate cell adhesion, inflammation and pyroptosis of HUVECs, therefore may also beneficial to attenuate AS progression. To further explore the mechanism herein, S1PR antagonist was added in TNF-α induced HUVECs before ApoM-S1P treatment. S1P regulates various biological activities by binding to different G-protein-coupled receptors known as S1PR1-S1PR5 [32]. Here, we observed that the S1PR2 antagonist reverse the protective effect of ApoM-S1P on pyroptosis and inflammation, presented by increased expressions of IL-1β, IL-18, IL-6 and IL-10. Therefore, we hypothesized that S1PR2 may have certain role to play in ApoM-S1P treatment to HUVECs. S1PR2 has potential utility in the drug targeted treatment of vascular inflammatory disorders [33]. Consistent with our hypothesis, data in previous study manifested that the pro-inflammatory impacts of S1P on HUVECs are largely mediated by S1PR2 [34]. The anti-inflammatory function of S1PR2 was also discovered in inflammatory bowel disease, as data showed that miR-126 could down-regulate S1PR2 expression and thus inhibit the activation of its downstream pathway PI3K/AKT signal pathway [35].

Although we found the implication of S1PR2 in pyroptosis of HUVECs, the possible downstream mechanism of S1PR2 in regulating pyroptosis of HUVECs remains to be clarified. PI3K/AKT is a downstream signaling pathway of S1P receptors, which is essential for vascular inflammation [21]. PI3K/AKT pathway-related proteins are the most frequently altered in human cancers, which associated with tumorigenesis, cellular transformation, drug resistance, and cancer progression [36]. Previous research has discovered that the alteration of PI3K/AKT pathway plays very important prognostic and predictive roles in colorectal cancer [37]. What’s more, growing evidences found that S1PR2 have a great impact on PI3K/AKT pathway. For instance, S1PR2 mediates endothelial cells dysfunction through PI3K/AKT pathway under high glucose condition [38]. The regulation of S1P/S1PR on AS progression is complicated and the protective role of ApoM-S1P on pyroptosis of HUVECs may have certain association with cellular signal pathway. S1PR2 has been found to associate with the G proteins Gα12/13, Gαq, or Gαi, while the coupling of S1PR2 with G proteins could further trigger the activation of PI3K/AKT pathway [39]. Although this study found the regulatory role of S1PR2 on p-PI3K and p-AKT levels, considering the complication of pathological courses of HUVEC injury and AS, we can only speculate a possible hypothesis and more studies are required to explore the possible mechanism herein. Due to time and budget limitations, we failed to set up an in vivo model to verify our speculation. But this indicates a promising future direction for our future studies.

## Conclusion

Collectively, in this study, the results concluded from above findings indicated that ApoM-S1P inhibits TNF-α-induced inflammation response in HUVECs through binding to S1PR2 to activate PI3K/AKT pathway. In this regards, we supported the beneficial role of ApoM-S1P in attenuating HUVECs injury. Nonetheless, these results must be interpreted with caution, and more studies are required to validate the results of current study.

## Data Availability

The datasets used or analyzed during the current study are available from the corresponding author on reasonable request.

## Abbreviations

ApoM: Apolipoprotein-M
ApoM-S1P: ApoM and S1P complex
AS: Atherosclerosis
ELISA: Enzyme-linked Immunosorbent assay
FBS: Fetal bovine serum
HDL: High-density lipoprotein
HUVECs: Human umbilical vein endothelial cells
qRT-PCR: Quantitative real-time polymerase chain reaction
ROS: Detection of reactive oxygen species
S1P: Sphingosine-1-phosphate
S1PR1: S1P receptor 1
